# Awareness of Lung Cancer Signs and Symptoms, Risk Factors, and Screening in Jordan: Cross-Sectional Study

**DOI:** 10.2196/86390

**Published:** 2026-05-19

**Authors:** Ahmad Ayman Yousef, Mohammad Yousef Saleh, Hani Shihadeh, Sanad Ahmad, Mo'tasem Alaiwah, Heba Shbailat, Karen Zaher Alsyouf, Malek Altaamreh, Eman Alshawish, Rasmieh Al amer

**Affiliations:** 1 School of Medicine University of Jordan Amman Jordan; 2 Clinical Nursing Department School of Nursing University of Jordan Amman Jordan; 3 Faculty of Nursing Pediatric Nursing and Midwifery Division An-Najah National University Nablus Occupied Palestinian Territory; 4 faculty of nursing Yarmouk University Irbid Jordan

**Keywords:** lung cancer awareness, lung cancer risk factors, lung cancer signs and symptoms, lung cancer screening, Jordan

## Abstract

**Background:**

Lung cancer is one of the most commonly diagnosed cancers worldwide and the leading cause of cancer-related mortality. Global studies have highlighted the importance of awareness of lung cancer signs and symptoms, as it influences health care seeking, diagnosis timing, and treatment outcomes. Despite the high prevalence of both lung cancer and smoking in Jordan, no studies have assessed lung cancer awareness among the Jordanian population. This study is the first in Jordan to reveal the levels of awareness of lung cancer signs and symptoms, risk factors, and screening and to identify factors associated with the awareness level.

**Objective:**

This study aimed to explore the awareness of lung cancer risk factors, signs and symptoms, and screening in the Jordanian population and the factors determining the levels of awareness.

**Methods:**

A cross-sectional survey was conducted across all 12 governorates of Jordan, including 498 adult participants. Data were collected using a structured questionnaire covering sociodemographic characteristics and awareness about lung cancer signs, symptoms, risk factors, and screening. Items were derived from the Lung Cancer Awareness Measure Toolkit, version 2.1, 2011. Awareness scores for lung cancer risk factors, signs and symptoms, and overall lung cancer awareness were calculated. Multivariate linear regression was used to identify significant predictors of awareness scores. Multivariate logistic regression was used to determine significant predictors of lung cancer screening awareness.

**Results:**

The mean lung cancer signs and symptoms awareness score was 5.4 (SD 4.9) out of 14, while the mean risk factor awareness score was 33.0 (SD 5.6) out of 45. The most recognized symptoms were coughing up blood (n=297, 59.6%) and pain upon breathing (n=237, 47.6%), while cigarette smoking (n=403, 80.9%) and exposure to air pollution (n=339, 68.1%) were the most recognized risk factors. Only 41.6% (n=207) of the participants had heard of lung cancer screening, while 79.3% (n=395) believed that screening improves survival. Being a smoker was predictive of lower awareness of lung cancer signs, symptoms, and risk factors, while a higher educational level and having a friend or relative previously diagnosed with lung cancer were associated with higher levels of awareness.

**Conclusions:**

This study revealed a low level of awareness of lung cancer signs and symptoms, risk factors, and screening in the Jordanian population. Further studies are needed to identify effective strategies for improving awareness, especially among high-risk populations.

## Introduction

According to recent statistics, lung cancer is one of the most commonly diagnosed cancers worldwide and the most common cause of cancer-related mortality [1]. Thus, it remains an extremely significant global health problem. In the Hashemite Kingdom of Jordan, lung cancer is also one of the most commonly diagnosed cancers and the most common cause of cancer-related mortality [1], which could be attributed to the very high tobacco smoking prevalence in the country [2]. Multiple studies have shown that low awareness of lung cancer signs and symptoms may be associated with longer delays in diagnosis [3-5], which in turn can lead to worse treatment outcomes and higher mortality rates [6]. In addition, research suggests that better awareness of lung cancer signs and symptoms and risk factors may lead to better attitudes toward smoking and alcohol habits [7]. This highlights the importance of promoting lung cancer awareness in different populations.

Several studies have evaluated awareness of lung cancer signs and symptoms and risk factors across various populations. In high-income nations, studies conducted in the United Kingdom, Australia, and Estonia revealed a low level of awareness. In all 3 studies, smoking was the most recognized risk factor, while shortness of breath and coughing up blood were the most recognized signs and symptoms. High socioeconomic status was predictive of higher awareness of signs, symptoms, and risk factors, while smokers had lower awareness levels than nonsmokers. Contradictory results were found regarding age and sex. In the United Kingdom and Australia, female participants had better awareness, while in Estonia, male participants demonstrated higher awareness. In the United Kingdom, older participants had better awareness, while in Australia and Estonia, younger people had better awareness [8-10]. Awareness of lung cancer signs, symptoms, and risk factors was also investigated in many low- and middle-income countries, including Nepal, Malaysia, India, and Nigeria. In concordance with results from high-income nations, tobacco smoking was the most commonly recognized risk factor, while other risk factors were poorly acknowledged, and coughing up blood was the most commonly recognized symptom [7,11-14].

Recently, research on awareness of lung cancer signs, symptoms, and risk factors has been conducted in several countries in the Middle East. In nations such as Syria, Saudi Arabia, and Palestine that are geographically and culturally similar to Jordan, results were also consistent with studies in other low- and middle-income nations. Overall levels of awareness were low, and higher levels of education and socioeconomic status were associated with higher levels of awareness [15-17]. The lack of such data in Jordan highlights the need for similar studies that explore the degree of awareness among the Jordanian population.

Considering the high prevalence and high mortality rate of lung cancer, along with the propensity for patients to seek medical attention at an advanced stage when treatment becomes more challenging [18], many resource-rich countries have implemented national screening programs for early detection of asymptomatic lung cancer using low-dose chest computed tomography scans. The survival benefit of lung cancer screening in populations at risk was confirmed by multiple randomized controlled trials, notably among individuals aged 50 to 80 years with a **≥**20-pack-year smoking history [19-21], highlighting the importance of lung cancer screening as a public health measure.

Many research efforts were directed toward investigating lung cancer screening awareness among both smokers and nonsmokers. In low- and middle-income nations, research in China found that only approximately 25% of high-risk individuals participated in lung cancer screening. Additionally, awareness of lung cancer screening benefits was low, with 72.1% of participants perceiving it as unnecessary [22]. Notably, Lin et al [23] reported that a higher level of awareness of lung cancer signs and symptoms was predictive of more positive lung cancer screening behaviors. Studies in Malaysia and Saudi Arabia revealed high rates of willingness to undergo screening (78.8%-94.7%) among high-risk individuals [17,24]. Despite the high rate of willingness to undergo lung cancer screening, significant barriers, including a lack of medical insurance or awareness of lung cancer risk factors, might limit participation in screening programs. In a high-income nation such as the United States, studies have also reported low levels of lung cancer screening awareness [25,26]. Various strategies designed to enhance awareness of lung cancer screening and perhaps increase participation rates in lung cancer screening programs have been examined. Multiple studies found that various educational programs successfully raised awareness of lung cancer screening and improved participation rates for lung cancer screening [27,28].

As per our knowledge, studies exploring awareness of lung cancer signs, symptoms, risk factors, and screening have not been conducted in Jordan, despite the high incidence of this cancer and the very high smoking prevalence. In addition, very few studies have investigated the factors associated with lung cancer screening awareness. The main aims of this study are to explore the levels of awareness of lung cancer signs and symptoms, risk factors, and screening in the Jordanian population and to explore the factors that predict these levels of awareness.

## Methods

### Study Design

This cross-sectional study was conducted across all 12 governorates of the Hashemite Kingdom of Jordan, covering both rural and urban regions, from March 2024 to September 2025. In total, 498 adult Jordanian citizens participated in the study. Due to limitations in time and resources, participants were recruited using convenience sampling. Data were collected through an online self-administered questionnaire distributed via Google Forms, which was shared on various social media platforms, including Facebook (Meta Platforms Inc) groups and WhatsApp (Meta Platforms Inc) channels. Only adult Jordanian citizens were eligible to complete the study. Individuals who were studying or working in health care–related fields were excluded from the study to reduce potential bias related to prior knowledge.

### Ethical Considerations

Ethics approval for this study was obtained from the institutional review board ethics committee at the University of Jordan (JU-2024/32). All participants provided electronic informed consent prior to completing the questionnaire. They were informed that all collected data would be collected anonymously, kept confidential, and stored securely in a nonidentifiable format. They were also informed that participation was voluntary and that they could withdraw from the study at any time. This study was conducted in accordance with institutional and national ethical standards and the Declaration of Helsinki (1975, updated 2013). Participants did not receive any compensations or financial incentives.

### Study Tool

Participants completed a questionnaire administered through Google Forms. The form consisted of 3 sections.

#### Section 1: Socioeconomic and Demographic Characteristics

This section gathered data on participants’ age, sex, place of residence, level of education, marital status, job, and smoking status.

#### Section 2: Awareness of Lung Cancer Signs, Symptoms, and Risk Factors

Items were adapted from the Cancer Research UK Lung Cancer Awareness Measure Toolkit, version 2.1. 2011, which had shown good validity and reliability [10]. The Arabic translation of the questionnaire followed the Chapman and Carter translation procedure [29], which included expert forward translation, review by bilingual experts, and cultural adaptation to ensure clarity and appropriateness.

The signs and symptoms awareness subscale included 14 items, with responses scored as 1 for “yes” and 0 for “no or don’t know.” Those who answered “yes” were deemed to recognize the sign or symptom. Included signs and symptoms were unexplained weight loss, persistent chest infection (**≥**3 weeks), persistent cough (2 to 3 weeks or longer), persistent tiredness or lack of energy, persistent shortness of breath, persistent chest pain, persistent shoulder pain, coughing up blood, ache or pain when breathing, loss of appetite, painful cough, finger or nail changes, a high-pitched sound when breathing, and progression or new development in an existing cough. The signs and symptoms awareness score was calculated by summing the scores across all 14 items. Cronbach α for the lung cancer signs and symptoms awareness subscale in this study was 0.936.

The lung cancer risk factor awareness subscale consisted of 9 items scored on a 5-point Likert scale (1=“strongly disagree,” 2=“disagree,” 3=“neutral or uncertain,” 4=“agree,” and 5=“strongly agree”). Participants who responded with “strongly agree” or “agree” were deemed to recognize the risk factor. Included risk factors were exposure to radon gas, exposure to passive smoking, previous cancer treatment, having a close relative with lung cancer, exposure to chemicals such as asbestos fibers, having a previous history of another cancer, such as head and neck cancer, exposure to air pollution, smoking, and having a history of chronic lung diseases such as chronic obstructive lung disease. A total risk factor awareness score was calculated for each participant by summing the scores across all 9 items, with higher scores indicating a higher level of awareness. Cronbach α for the lung cancer risk factors awareness subscale in this study was 0.901.

A total lung cancer awareness score was calculated by summing the total signs and symptoms awareness score and the total risk factors awareness score.

#### Section 3: Awareness of Lung Cancer Screening

Participants were asked whether they had ever heard about lung cancer screening and whether they believed that early detection of lung cancer improves cure and survival rates.

### Data Analysis

Statistical analysis was performed using SPSS (version 27.0.1; IBM Corp). Descriptive statistics were used to summarize the characteristics of the study population. Inferential statistical tests, including 2-tailed independent samples *t* tests and 1-way ANOVA, were used to compare mean awareness scores across demographic subgroups. Multivariate linear regression was conducted to identify significant predictors of awareness scores related to lung cancer signs, symptoms, and risk factors, while multivariate logistic regression analysis was used to determine the predictors of awareness related to lung cancer screening. A *P* value of <.05 was considered statistically significant for all analyses.

## Results

### Descriptive Statistics

Table 1 summarizes the descriptive statistics of the study participants. The mean age of participants was 35.2 (SD 13.2) years. Most participants resided in an urban area, and most had completed either high school or a bachelor’s degree or diploma. Overall, 39.6% (n=197) of participants were active smokers. The mean lung cancer risk factors awareness score was 33.0 (SD 5.6, range 9-45). The mean lung cancer signs and symptoms awareness score was 5.4 (SD 4.9, range 0-14). The mean total lung cancer awareness score was 38.4 (SD 8.4, range 9-59). In total, 41.6% (n=207) of the study participants had ever heard of lung cancer screening, while 79.3% (n=395) believed that lung cancer screening for early detection improves survival.

**Table 1:** Descriptive statistics of the study participants (N=498).

Variable			Values
Age (years), mean (SD)			35.2 (13.2)
**Sex, n (%)**			
	Female	314 (63.1)	
	Male	184 (36.9)	
**Place of residence, n (%)**			
	Urban	449 (90.2)	
	Rural	49 (9.8)	
**Educational level, n (%)**			
	Elementary school	38 (7.6)	
	High school	74 (14.9)	
	Bachelor’s degree or diploma	337 (67.7)	
	Postgraduate	49 (9.8)	
**Marital status, n (%)**			
	Single	200 (40.2)	
	Married	298 (59.8)	
**Smoking status, n (%)**			
	Nonsmoker	301 (60.4)	
	Smoker	197 (39.6)	
**Lung cancer risk factors awareness score^a^**			
	Mean (SD)	33.0 (5.6)	
	Median (IQR)	33.0 (29.0-36.0)	
**Lung cancer signs and symptoms awareness score^b^**			
	Mean (SD)	5.4 (4.9)	
	Median (IQR)	4.0 (0.0-10.0)	
**Total lung cancer awareness score^c^**			
	Mean (SD)	38.4 (8.4)	
	Median (IQR)	38.0 (32.0-44.0)	
“**Have you ever heard of lung cancer screening for early detection?” n (%)**			
	Yes	207 (41.6)	
	No	291 (58.4)	
“**Do you think that lung cancer screening for early detection can improve survival?” n (%)**			
	Yes	395 (79.3)	
	No or do not know	103 (20.7)	

^a^Range for possible lung cancer risk factors awareness scores was 9-45.

^b^Range for possible lung cancer signs and symptoms awareness scores was 0-14.

^c^Range for possible total lung cancer awareness scores was 9-59.

### Recognition of Lung Cancer Signs and Symptoms and Risk Factors

Table 2 summarizes the frequencies of recognition of the different lung cancer signs, symptoms, and risk factors. The most recognized signs and symptoms were coughing up blood (n=297, 59.6%) and pain when breathing (n=237, 47.6%), while the least recognized signs and symptoms were persistent shoulder pain (n=108, 21.7%) and changes in the shape of fingers or nails (n=122, 24.5%). The most recognized risk factors were being a smoker (n=403, 80.9%) and exposure to air pollution (n=339, 68.1%), while the least recognized risk factors were receiving treatment for a previous cancer (n=175, 35.1%) and having a past history of cancer (n=204, 41%).

**Table 2:** Recognition of signs, symptoms, and risk factors of lung cancer.

Variable			Values, n (%)
**Symptoms**			
	Unexplained weight loss	152 (30.5)	
	Persistent chest infection	195 (39.2)	
	Cough that does not go away	168 (33.7)	
	Shortness of breath	224 (45)	
	Persistent tiredness	168 (33.7)	
	Persistent chest pain	216 (43.4)	
	Persistent shoulder pain	108 (21.7)	
	Coughing up blood	297 (59.6)	
	Ache or pain when breathing	237 (47.6)	
	Loss of appetite	176 (35.3)	
	Painful cough	228 (45.8)	
	Changes in the shape of the fingernails	122 (24.5)	
	High-pitched sound when breathing	205 (41.2)	
	Change in existing cough	203 (40.8)	
**Risk factors**			
	Exposure to radon gas	233 (46.8)	
	Passive cigarette smoke exposure	326 (65.5)	
	Past cancer treatment	175 (35.1)	
	Close relative with lung cancer	219 (44)	
	Exposure to chemicals (eg, asbestos)	249 (50)	
	Past history of cancer (eg, head and neck cancer)	204 (41)	
	Exposure to air pollution	339 (68.1)	
	Being a smoker	403 (80.9)	
	History of lung disease (eg, chronic obstructive pulmonary disease)	337 (67.7)	

### Association Among Risk Factors Awareness Score, Signs and Symptoms Awareness Score, Total Lung Cancer Awareness Score, and Demographic Characteristics

Table 3 summarizes lung cancer risk factors awareness score, signs and symptoms awareness score, and total lung cancer awareness score across the different demographic categories. Participants aged <35 years had a significantly higher level of awareness of lung cancer signs and symptoms (*P*=.04). Smokers had a significantly lower level of awareness of both lung cancer risk factors (*P*<.001) and signs and symptoms (*P*=.003). No significant differences in awareness of lung cancer risk factors and symptoms were observed across sex, place of residence, level of education, and marital status categories.

**Table 3:** Comparison of risk factors awareness scores, signs and symptoms awareness scores, and total lung cancer awareness scores across demographic categories.

Variable		Risk factors awareness score^a^				Signs and symptoms awareness score^b^					Total lung cancer awareness score^c^		
		Mean (SD)	*P* value		Mean (SD)		*P* value		Mean (SD)			*P* value	
**Age (years)**				.34				.04					.06
	≥35	32.7 (4.6)			5.0 (5.0)				37.7 (7.9)				
	<35	33.2 (6.5)			5.9 (4.8)				39.1 (8.9)				
**Gender**				.18				.54					.19
	Female	33.3 (4.7)			5.5 (4.7)				38.8 (7.7)				
	Male	32.5 (6.9)			5.2 (5.4)				37.7 (9.4)				
**Place of residence**				.74				.15					.28
	Rural	33.2 (6.5)			6.4 (5.2)				39.6 (9.3)				
	Urban	32.9 (5.5)			5.3 (4.9)				38.3 (8.3)				
**Level of education**				.49				.17					.14
	Elementary school	31.7 (6.4)			3.9 (5.2)				35.6 (9.6)				
	High school	32.8 (4.9)			5.0 (5.1)				37.8 (8.2)				
	Bachelor’s degree or diploma	33.1 (5.7)			5.7 (4.9)				38.8 (8.2)				
	Postgraduate	33.3 (5.7)			5.6 (4.9)				38.9 (8.6)				
**Marital status**				.73				.33					.42
	Single	33.1 (6.7)			5.7 (4.9)				38.8 (9.1)				
	Married	32.9 (4.7)			5.2 (5.0)				38.1 (7.9)				
**Smoking status**				<.001				.003					<.001
	Nonsmoker	33.9 (5.1)			6.0 (4.7)				39.9 (7.8)				
	Smoker	31.6 (6.1)			4.6 (5.2)				36.2 (8.9)				

^a^Range for possible risk factors awareness scores was 9-45.

^b^Range for possible signs and symptoms awareness scores was 0-14.

^c^Range for possible total lung cancer awareness scores was 9-59.

### Linear Regression Analysis

Table 4 summarizes the results of linear regression analysis used to predict risk factors awareness score, signs and symptoms awareness score, and total lung cancer awareness score. Having a bachelor’s degree or diploma level of education was significantly associated with a higher lung cancer signs and symptoms awareness score (β=1.89, 95% CI 0.23-3.55; *P*=.03). Being a smoker was significantly predictive of a lower total lung cancer awareness score (β=−3.74, 95% CI −5.29-−2.19; *P*<.001) as well as lower scores in both the risk factors (β=−2.23, 95% CI −3.29-−1.17; *P*<.001) and signs and symptoms (β=−1.51, 95% CI −2.42-−0.59; *P*<.001) domains. Having a friend or relative diagnosed with lung cancer was significantly predictive of higher symptom awareness scores (β=2.74, 95% CI 1.79-3.68; *P*<.001) and total lung cancer awareness scores (β=3.69, 95% CI 2.08-5.29; *P*<.001). There was no significant association between age, gender, place of residence, educational level, marital status, or having friends or relatives working in the health care sector, and total lung cancer awareness score.

**Table 4:** Predictors of lung cancer risk factors awareness score, signs and symptoms awareness score, and total lung cancer awareness score (multivariate linear regression analysis).

Predictor variable			Risk factors awareness score^a^				Signs and symptoms awareness score^b^				Total lung cancer awareness score^c^		
			β coefficient (95% CI)		*P* value		β coefficient (95% CI)		*P* value		β coefficient (95% CI)		*P* value
Age (years)			−0.04 (−0.10-0.01)		.13		−0.01 (−0.05-0.04)		.84		−0.05 (−0.13-0.03)		.25
**Sex**													
	Female	Reference		Reference		Reference		Reference		Reference		Reference	
	Male	0.11 (−1.09-1.11)		.98		0.36 (−0.60-1.31)		.46		0.37 (−1.24-1.98)		.65	
**Place of residence**													
	Rural	Reference		Reference		Reference		Reference		Reference		Reference	
	Urban	−0.28 (−1.96-1.40)		.74		−1.28 (−2.74-0.18)		.08		−1.56 (−4.03-0.91)		.22	
**Educational level**													
	Elementary school	Reference		Reference		Reference		Reference		Reference		Reference	
	High school	0.64 (−1.54-2.83)		.56		0.93 (−0.96-2.82)		.33		1.58 (−1.63-4.78)		.33	
	BSc or diploma	0.71 (−1.21-2.63)		.47		1.89 (0.23-3.55)		.03		2.60 (−0.22-5.42)		.07	
	Postgraduate	0.47 (−1.94-2.88)		.70		1.47 (−0.61-3.56)		.17		1.94 (−1.59-5.48)		.28	
**Marital status**													
	Single	Reference		Reference		Reference		Reference		Reference		Reference	
	Married	0.66 (–0.80-2.12)		.37		−0.18 (–1.44-1.09)		.79		0.49 (–1.66-2.63)		.66	
**Smoking status**													
	Nonsmoker	Reference		Reference		Reference		Reference		Reference		Reference	
	Smoker	−2.23 (−3.29-−1.17)		<.001		−1.51 (−2.42-−0.59)		<.001		−3.74 (−5.29-−2.19)		<.001	
“**Do you have any friends or relatives who work in the health care sector?”**													
	No	Reference		Reference		Reference		Reference		Reference		Reference	
	Yes	0.65 (–0.71-2.01)		.35		−0.35 (–1.53-0.83)		.56		0.31 (–1.69-2.30)		.76	
“**Have you ever had a friend or a relative who was diagnosed with lung cancer?”**													
	No	Reference		Reference		Reference		Reference		Reference		Reference	
	Yes	0.95 (–0.14-2.05)		.09		2.74 (1.79-3.68)		<.001		3.69 (2.08-5.29)		<.001	

^a^Range for possible risk factors awareness scores was 9-45. *R*^2^=0.054; adjusted *R*^2^=0.034; *F*_10,485_=2.75; *P*=.003. All variance inflation factor values were <5, indicating an acceptable degree of collinearity.

^b^Range for possible signs and symptoms awareness scores was 0-14. *R*^2^=0.093; adjusted *R*^2^=0.074; *F*_10,485_*=*4.96; *P*<.001. All variance inflation factor values were <5, indicating an acceptable degree of collinearity.

^c^Range for possible total lung cancer awareness scores was 9-59. *R*^2^=0.095; adjusted *R*^2^=0.076; *F*_10,485_=5.05; *P*<.001. All variance inflation factor values were <5, indicating an acceptable degree of collinearity.

### Logistic Regression Analysis

Table 5 summarizes the results of logistic regression analysis used to determine the factors predictive of the odds of recognition of lung cancer screening and believing that screening for early detection improves survival. Having a friend or relative diagnosed with lung cancer (odds ratio 2.90, 95% CI 1.87- 4.48; *P*<.001) and a higher symptom awareness score (odds ratio 1.10, 95% CI 1.06-1.15; *P*<.001) were significantly associated with higher odds of recognition of lung cancer screening. Having a higher educational level (Bachelor’s degree or diploma: odds ratio 2.73, 95% CI 1.23-6.07; *P*=.01 vs postgraduate odds ratio 3.96, 95% CI 1.28-12.25; *P*=.02), a higher symptom awareness score (odds ratio 1.14, 95% CI 1.07-1.21; *P*<.001), and a higher risk factor awareness score (odds ratio 1.08, 95% CI 1.03-1.13; *P*<.001) were significantly associated with higher odds of believing that lung cancer screening is effective for early diagnosis and improving survival.

**Table 5:** Factors associated with the odds of recognition of lung cancer screening and the belief that early detection improves survival in patients with lung cancer (logistic regression analysis).

Predictor variables		“Have you ever heard of lung cancer screening for early detection?”^a^		“Do you think that lung cancer screening for early diagnosis improves survival in patients with lung cancer?”^b^	
		Odds ratio (95% CI)	*P* value	Odds ratio (95% CI)	*P* value
Age (years)		1.01 (0.99-1.03)	.27	1.00 (0.98-1.03)	.95
**Gender**					
	Female	Reference	Reference	Reference	Reference
	Male	1.38 (0.90-2.12)	.14	0.88 (0.52-1.48)	.62
**Place of residence**					
	Rural	Reference	Reference	Reference	Reference
	Urban	1.23 (0.63-2.41)	.55	0.58 (0.23-1.43)	.23
**Educational level**					
	Elementary school	Reference	Reference	Reference	Reference
	High school	0.73 (0.30-1.73)	.47	2.51 (1.00-6.32)	.05
	Bachelor’s degree or diploma	0.96 (0.45-2.06)	.91	2.73 (1.23-6.07)	.01
	Postgraduate	1.23 (0.48-3.16)	.67	3.96 (1.28-12.25)	.02
**Marital status**					
	Single	Reference	Reference	Reference	Reference
	Married	0.65 (0.36-1.15)	.14	0.99 (0.50-1.99)	.99
**Smoking status**					
	Nonsmoker	Reference	Reference	Reference	Reference
	Smoker	1.02 (0.67-1.57)	.93	1.20 (0.71-2.01)	.50
“**Have you ever had any friends or relatives who were diagnosed with lung cancer?”**					
	No	Reference	Reference	Reference	Reference
	Yes	2.90 (1.87-4.48)	<.001	1.28 (0.72-2.28)	.40
“**Do you have any friends or relatives who work in the health care sector?”**					
	No	Reference	Reference	Reference	Reference
	Yes	1.25 (0.72-2.17)	.43	1.07 (0.57-2.00)	.84
Risk factors awareness score^c^		1.01 (0.97-1.04)	.74	1.08 (1.03-1.13)	<.001
Signs and symptoms awareness score^d^		1.10 (1.06-1.15)	<.001	1.14 (1.07-1.21)	<.001

^a^Yes=1 and no=0. Hosmer-Lemeshow test: *P*=.82.

^b^Yes=1 and no or do not know=0. Hosmer-Lemeshow test: *P*=.05.

^c^Range for possible risk factors awareness scores was 9-45.

^d^Range for possible signs and symptom awareness scores was 0-14.

No significant association was found between age, gender, residence, marital status, smoking status, or having friends or family in the health care profession and the recognition of lung cancer screening or believing that screening is effective for enhancing survival through early diagnosis.

## Discussion

### Principal Findings

The primary objective of this study was to assess population-level awareness of lung cancer signs, symptoms, risk factors, and screening in Jordan. Our study revealed a lung cancer signs and symptoms awareness score of 5.4, indicating that, on average, participants could recognize approximately 5 to 6 signs or symptoms of lung cancer, reflecting a low level of awareness despite the relatively high incidence of lung cancer in Jordan. This score is comparable to the score observed in Nigeria (5) [13] but is significantly lower than the scores reported in the United Kingdom (8.78) [10] and Syria (8.63) [15]. This could be attributed to the lack of national awareness initiatives focused on lung cancer in Jordan and the nonspecific nature of lung cancer signs and symptoms, which could be easily confused with symptoms or signs of other lung diseases that are prevalent in the country (eg, chronic obstructive pulmonary disease). The mean risk factor awareness score was 33.0 (SD 5.6), which is comparable to that reported in the United Kingdom (35.6) [10].

Smokers had a significantly lower level of awareness of both lung cancer risk factors (*P*<.001) and signs and symptoms (*P*=.003), aligning with most existing literature [8-10,15]. This association might reflect multiple factors. Smokers might be more likely to avoid or disengage from health awareness–promoting programs due to perceived threat or denial. In addition, tobacco smoking may be more common in populations with lower socioeconomic status and educational attainment, which might also limit their awareness. This finding elucidates the importance of focusing awareness programs on smokers due to their increased susceptibility to lung cancer.

Younger participants had greater awareness of lung cancer signs and symptoms, consistent with studies from Australia [8], Estonia [9], and Saudi Arabia [17], but divergent from findings from the United Kingdom [10], where older individuals exhibited greater awareness. This could be attributed to younger populations having greater access to social media and online information platforms, which promote their health awareness. However, in other countries, cancer awareness programs might focus on older populations due to their increased susceptibility.

Having a friend or relative diagnosed with lung cancer was significantly associated with a higher lung cancer signs and symptoms awareness score (β=2.74; *P*<.001), highlighting the influence of personal connections on an individual’s perception of their own signs and symptoms.

Consistent with studies from high-, low-, and middle-income countries, smoking was the most recognized risk factor for lung cancer in Jordan, reflecting the global success of antitobacco campaigns in raising awareness of smoking as the leading preventable cause of lung cancer. Other risk factors (eg, exposure to asbestos fibers, radon gas, and receiving treatment for a previous cancer) were much less recognizable, likely due to their relative rarity compared to tobacco smoking. This highlights the importance of focusing health awareness programs on the most overlooked risk factors, particularly in populations with distinct exposures (eg, individuals whose occupations involve asbestos exposure).

Signs and symptoms recognition was variable across different countries. In Jordan and the United Kingdom, the most recognized symptom was coughing up blood [10], while shortness of breath was the most recognized symptom in Syria [15], Saudi Arabia [17], and Australia [8]. Change in a pre-existing cough and prolonged cough were the most commonly recognized in Palestine and Estonia, respectively [9,16]. This variability could be attributed to the varying prevalence of other respiratory diseases across the globe. In populations with a high prevalence of tuberculosis, for example, coughing up blood may be less recognized as a symptom of lung cancer, while in populations with a high prevalence of chronic obstructive pulmonary disease, shortness of breath and a change in a pre-existing cough may be less perceived as serious.

The least recognized lung cancer signs and symptoms in our study were persistent shoulder pain and changes in the shape of the nails. Unlike the more prominent respiratory symptoms (eg, coughing up blood), these symptoms may be seen as unrelated to the lungs in common perception; rather, they might be linked to the musculoskeletal system.

A small proportion of participants (n=207, 41.6%) in our study recognized the concept of lung cancer screening, while the majority (n=395, 79.3%) believed that screening improves survival. These results are comparable to findings from the United States [25], where 44% of participants had heard of and were aware of lung cancer screening, and 93% believed that catching it early results in better outcomes. The high proportion of participants believing that screening for early detection improves survival despite the low level of lung cancer screening recognition might be due to the common misconception that early diagnosis improves outcomes in all cancers, while the low level of recognition of lung cancer screening might be due to its relative novelty compared to screening for other cancers (eg, breast cancer). Having a friend or relative diagnosed with lung cancer was associated with higher odds of recognition of lung cancer screening, again highlighting the importance of close people’s experiences in perceptions of an individual’s own health.

Higher lung cancer awareness scores were significantly associated with higher odds of recognizing lung cancer screening and believing that it improves survival, highlighting the potential role of improving lung cancer awareness in promoting positive attitudes toward lung cancer screening.

To the best of our knowledge, this is the first study to assess population awareness of lung cancer symptoms, risk factors, and screening in the Hashemite Kingdom of Jordan, and this is the first study to identify and quantify that having a relative or friend diagnosed with lung cancer increases awareness of lung cancer signs, symptoms, and screening. Further research should be done to identify effective interventions to improve knowledge and awareness of lung cancer, minimize diagnostic delay, and improve early detection and participation rates in lung cancer screening, especially among high-risk groups. In addition, further studies are warranted to explore characteristics of patients with lung cancer in Jordan, including involvement in screening programs, stage at diagnosis, and unique exposures.

### Limitations

Several limitations should be acknowledged when considering the findings of our study. The use of an online form to collect data excludes individuals with no access to social media or the internet, which might induce selection bias, resulting in an overestimation of awareness levels. This approach may also have introduced bias toward younger participants, as they are more likely to use social media, thereby limiting the representation of older adults who are at higher risk of developing lung cancer. The small sample size limits the generalizability and the representativeness of our sample. Participants’ governorates of residence were not recorded, limiting the ability to determine geographic representativeness and identify potential regional variation in lung cancer awareness across Jordan. The reliance on a self-administered questionnaire and the lack of concise explanations limited deeper exploration of participants’ understanding. Moreover, the inability to completely exclude health care professionals might bias the results toward higher awareness levels. Our study had a pure quantitative design and lacked a qualitative approach to evaluate lung cancer awareness, depended on recognition rather than recall, and used closed-ended rather than open-ended questions. Our study included Jordanian adults of all ages, with no focus on older people despite their higher susceptibility.

### Conclusions

Our study revealed a low level of awareness of lung cancer signs and symptoms, risk factors, and screening. Higher awareness was associated with younger age, having a friend or relative diagnosed with lung cancer, while smoking was linked with a lower level of awareness. These findings underscore the importance of implementing targeted, age-inclusive public health strategies to improve lung cancer awareness and promote early detection, particularly among high-risk groups.

## Data Availability

The data supporting the findings of this study are available from the corresponding author upon reasonable request.
